# Synchronous and Metachronous Peritoneal Metastases in Patients with Left-Sided Obstructive Colon Cancer

**DOI:** 10.1245/s10434-020-08327-7

**Published:** 2020-03-13

**Authors:** Joyce Valerie Veld, Daniel Derk Wisselink, Femke Julie Amelung, Esther Catharina Josephina Consten, Johannes Hendrik Willem de Wilt, Ignace de Hingh, Wilhelmus Adrianus Bemelman, Jeanin Elise van Hooft, Pieter Job Tanis, Dutch Snapshot Research Group, H. Algera, G. D. Algie, C. S. Andeweg, T. E. Argillander, M. N. N. J. Arron, K. Arts, T. H. J. Aufenacker, I. S. Bakker, M. van Basten Batenburg, A. J. N. M. Bastiaansen, G. L. Beets, A. van den Berg, B. van de Beukel, R. L. G. M. Blom, B. Blomberg, E. G. Boerma, F. C. den Boer, W. A. A. Borstlap, N. D. Bouvy, J. E. Bouwman, N. D. A. Boye, A. R. M. Brandt-Kerkhof, H. T. Bransma, A. Breijer, W. T. van den Broek, M. E. E. Bröker, J. P. M. Burbach, E. R. J. Bruns, T. A. Burghgraef, R. M. P. H. Crolla, M. Dam, L. Daniels, J. W. T. Dekker, A. Demirkiran, K. W. van Dongen, S. F. Durmaz, A. van Esch, J. A. van Essen, P. Fockens, J. W. Foppen, E. J. B. Furnee, A. A. W. van Geloven, M. F. Gerhards, E. A. Gorter, W. M. U. van Grevenstein, J. van Groningen, I. A. J. de Groot-van Veen, H. E. Haak, J. W. A. de Haas, P. van Hagen, E. E. van Halsema, J. T. H. Hamminga, K. Havenga, B. van den Hengel, E. van der Harst, J. Heemskerk, J. Heeren, B. H. M. Heijnen, L. Heijnen, J. T. Heikens, M. van Heinsbergen, D. A. Hess, N. Heuchemer, C. Hoff, W. Hogendoorn, A. P. J. Houdijk, N. Hugen, B. Inberg, T. L. Janssen, D. Jean Pierre, W. J. de Jong, A. C. H. M. Jongen, A. V. Kamman, J. M. Klaase, W. Kelder, E. F. Kelling, R. Klicks, G. W. De Klein, F. W. H. Kloppenberg, J. L. M. Konsten, L. J. E. R. Koolen, V. Kornmann, R. T. J. Kortekaas, A. Kreiter, B. Lamme, J. F. Lange, T. Lettinga, D. Lips, G. Lo, F. Logeman, Y. T. van Loon, M. F. Lutke Holzik, C. C. M. Marres, I. Masselink, A. Mearadji, G. Meisen, A. G. Menon, J. W. S. Merkus, D. J. L. M. de Mey, H. C. J. van der Mijle, D. E. Moes, C. J. L. Molenaar, M. J. Nieboer, K. Nielsen, G. A. P. Nieuwenhuijzen, P. A. Neijenhuis, P. Oomen, N. van Oorschot, K. Parry, K. C. M. J. Peeters, T. Paulides, I. Paulusma, F. B. Poelmann, S. W. Polle, P. Poortman, M. H. Raber, R. J. Renger, B. M. M. Reiber, R. Roukema, W. M. J. de Ruijter, M. J. A. M. Russchen, H. J. T. Rutten, J. Scheerhoorn, S. Scheurs, H. Schippers, V. N. E. Schuermans, H. J. Schuijt, P. D. Siersema, J. C. Sierink, C. Sietses, R. Silvis, J. van der Slegt, G. D. Slooter, M. van der Sluis, P. van der Sluis, N. Smakman, D. Smit, A. B. Smits, T. C. van Sprundel, D. J. A. Sonneveld, C. Steur, J. Straatman, M. C. Struijs, H. A. Swank, A. K. Talsma, M. Tenhagen, F. ter Borg, J. A. M. G. Tol, J. L. Tolenaar, L. Tseng, J. B. Tuynman, M. J. F. van Veen, S. C. Veltkamp, A. W. H. van de Ven, L. Verkoele, M. Vermaas, H. P. Versteegh, L. Verslijs, T. Visser, D. van Uden, W. J. Vles, R. J. de Vos tot Nederveen Cappel, H. S. de Vries, S. T. van Vugt, G. Vugts, J. A. Wegdam, T. J. Weijs, B. J. van Wely, M. Westerterp, H. L. van Westreenen, B. Wiering, N. A. T. Wijffels, A. A. Wijkmans, L. H. Wijngaarden, M. van de Wilt, F. Wit, E. S. van der Zaag, D. D. E. Zimmerman, T. L. R. Zwols

**Affiliations:** 1 Department of Surgery, Amsterdam University Medical Centers, University of Amsterdam, Cancer Centre Amsterdam, Amsterdam, The Netherlands; 2 Department of Gastroenterology and Hepatology, Amsterdam University Medical Centers, University of Amsterdam, Cancer Centre Amsterdam, Amsterdam, The Netherlands; 3 Department of Surgery, Meander Medical Center, Amersfoort, The Netherlands; 4 Department of Surgery, University Medical Center Utrecht, Utrecht, The Netherlands; 5 Department of Surgery, University Medical Center Groningen, Groningen, The Netherlands; 6 Department of Surgery, Radboud University Medical Center, Nijmegen, The Netherlands; 7 Department of Surgery, Catharina Hospital, Eindhoven, The Netherlands

## Abstract

**Background:**

Controversy exists on emergency setting as a risk factor for peritoneal metastases (PM) in colon cancer patients. Data in patients with obstruction are scarce. The aim of this study was to determine the incidence of synchronous and metachronous PM, risk factors for the development of metachronous PM, and prognostic implications within a large nationwide cohort of left-sided obstructive colon cancer (LSOCC).

**Methods:**

Patients with LSOCC treated between 2009 and 2016 were selected from the Dutch ColoRectal Audit. Additional treatment and long-term outcome data were retrospectively collected from original patient files in 75 hospitals in 2017.

**Results:**

In total, 3038 patients with confirmed obstruction and without perforation were included. Synchronous PM (at diagnosis or < 30 days postoperatively) were diagnosed in 148/2976 evaluable patients (5.0%), and 3-year cumulative metachronous PM rate was 9.9%. Multivariable Cox regression analyses revealed pT4 stage (HR 1.782, 95% CI 1.191–2.668) and pN2 stage (HR 2.101, 95% CI 1.208–3.653) of the primary tumor to be independent risk factors for the development of metachronous PM. Median overall survival in patients with or without synchronous PM was 20 and 63 months (*p* < 0.001) and 3-year overall survival of patients that did or did not develop metachronous PM was 48.1% and 77.0%, respectively (*p* < 0.001).

**Conclusion:**

This population based study revealed a 5.0% incidence of synchronous peritoneal metastases in patients who underwent resection of left-sided obstructive colon cancer. The subsequent 3-year cumulative metachronous PM rate was 9.9%, with advanced tumor and nodal stage as independent risk factors for the development of PM.

**Electronic supplementary material:**

The online version of this article (10.1245/s10434-020-08327-7) contains supplementary material, which is available to authorized users.

Colorectal cancer is the third most common malignancy worldwide. In these patients, the peritoneum is the second most common place of recurrence.^1^^,^^2^ Published incidence rates of metachronous PM are influenced by characteristics of the colorectal cancer population, as well as the method of detection. Sensitivity of imaging is low for the small flat peritoneal lesions, and metachronous PM might remain undetected unless surgical re-exploration is performed.^3^ Consequently, incidences may be underestimated. Prognosis is generally poor at time of diagnosis, with a median survival of approximately 5 months when untreated.^4^^,^^5^

Published risk factors for the development of metachronous PM in colorectal cancer are advanced tumor (T) and nodal (N) status, mucinous histology, emergency surgery, and non-radical resection of the primary tumor.^6^^,^^7^ Emergency surgery is mostly performed for either tumor perforation, obstruction, or bowel perforation proximal to an obstructing cancer. In a systematic review by Honoré et al., tumor perforation was identified as a risk factor for metachronous PM. Regarding obstruction, the authors stated that no increased risk for the development of metachronous PM was reported in 12 large series, although this association was not part of the aims of these studies.^8^ The authors updated their review in 2017, and no new data to modify their conclusion on obstruction as a risk factor for peritoneal recurrence was available.^9^

In 2017, a nationwide collaborative research project on left-sided obstructing colon cancer (LSOCC) was performed in the Netherlands.^10^ Given the paucity of data on PM in patients with obstructing colon cancer, the primary aim of this study was to determine the incidence of synchronous PM and cumulative metachronous PM rate using this large dataset. Secondary objectives were to provide independent predictors of metachronous PM in this patient population, and to evaluate therapeutic and prognostic implications.

## Methods

### Study Design

A collaborative, national research project was performed by the Dutch Snapshot Research Group (DSRG) according to a previously published protocol.^10^ Short-term data of patients with a registered resection of LSOCC between 2009 and 2016 were retrieved from the Dutch Colorectal Audit (DCRA). In this national, prospective (mandatory) database, all patients with colorectal cancer had undergone either emergency or elective surgical resection for primary colorectal cancer. Left-sided resections were defined as resection for a tumor located in the splenic flexure, descending colon or sigmoid. Additional baseline, procedural, and long-term outcome data were retrospectively gathered from original patient files by surgical residents between August and December 2017.^11^ The design of this study and preparation of the manuscript were performed according to the Strengthening The Reporting of Observational Studies in Epidemiology (STROBE) guidelines.^12^

### Inclusion/Exclusion Criteria

After collection of additional diagnostic data from the original patient files, only patients with a documented symptomatic colonic obstruction with complaints of either nausea, vomiting, and/or abdominal distention with confirmation of the obstruction on X-ray or computed tomography (CT) were considered as a diagnosis of LSOCC. Patients without proven malignancy, an extracolonic malignancy, and/or signs of bowel perforation on CT at baseline were excluded.

### Outcome Parameters and Definitions

The main outcomes of this study were the incidence of synchronous PM and cumulative metachronous PM rate. Secondary outcomes included risk factors for the development of metachronous PM, and treatment of PM.

Synchronous PM were defined as PM present at time of diagnosis or observed within 30 days after resection of the primary tumor according to Segelman et al.^6^ Metachronous PM included PM observed after 30 days following primary tumor resection. For analyses of synchronous PM, all patients were included independent of intention of treatment. For analyses of metachronous PM, patients were excluded if they had synchronous PM, palliative treatment intent post-resection, palliative treatment intent based on review of original patient files, or if patients died within 30 days postoperatively. For synchronous PM, overall survival included the interval between first presentation and death by any cause, or last follow-up. For metachronous PM, overall survival was defined as the interval from primary tumor resection until death by any cause or last follow-up. Treatment was categorized as cytoreductive surgery and hyperthermic intraperitoneal chemotherapy (CRS/HIPEC) or other modalities.

### Statistical Analysis

Normally distributed continuous outcomes are reported as means with standard deviation (SD) and analysed with Student’s *t* test. Non-normally distributed continuous data are shown as medians with interquartile range (IQR) and compared with the Mann–Whitney *U* test. Categorical variables are presented as percentages and compared with the *X*^2^ test or Fisher’s exact test. Kaplan–Meier analysis was used to determine the cumulative metachronous PM rate. To determine independent risk factors for the development of metachronous PM, Cox regression analyses were performed. Covariates were included in the univariable Cox regression analysis based on previous literature in combination with initial analyses of baseline and procedural characteristics. Covariates with a *p*-value of < 0.2 after univariable analysis were included in the multivariable Cox regression model to identify individual risk factors for the development of metachronous PM. Overall survival was calculated and plotted using Kaplan–Meier analysis for the different predefined subgroups and compared using the log-rank test. A two-sided *p* value of < 0.05 was considered statistically significant. Analyses were performed with IBM SPSS statistics, version 25.0 (IBM Corp Amonk, NY, USA).

### Ethics

The Institutional Review Board of the Academic Medical Center in Amsterdam (the Netherlands) approved this study, with exemption status for individual informed consent because of the retrospective, anonymized data analyses.

## Results

### Demographics

**Fig. 1:**
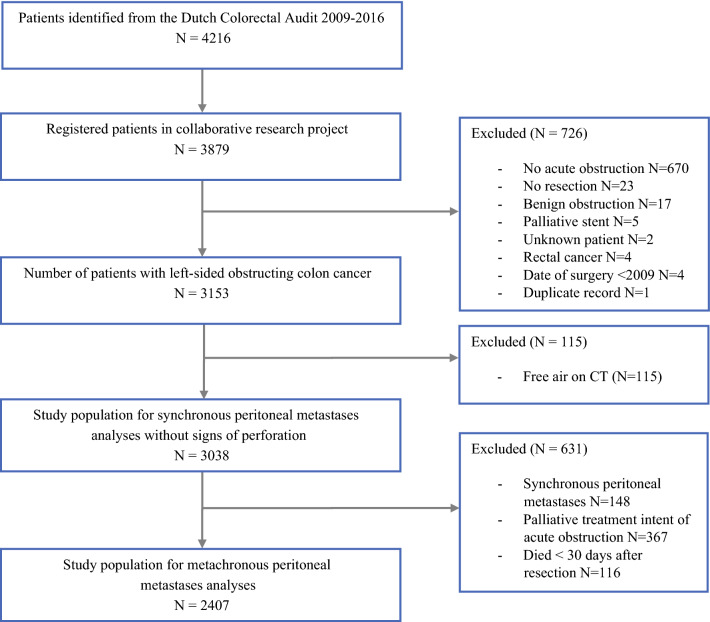
Patient selection

Of the 77 hospitals in the Netherlands in 2017, 75 hospitals participated, resulting in a registration of 3879 potentially eligible patients (Fig. 1). After applying strict inclusion criteria, mainly related to a confirmed diagnosis of acute colonic obstruction without signs of bowel perforation, 3038 patients remained for analysis on synchronous PM. A total of 2407 patients were included for analyses on metachronous PM, after exclusion of patients with synchronous PM (*N* = 148), patients with palliative intention of treatment (*N* = 367), and patients who died within 30 days after resection (*N* = 116).

### Synchronous Peritoneal Metastases

#### Baseline and Procedural Characteristics

Presence of PM at diagnosis of the primary tumor or until 30 days postoperatively was missing in 62 of 3038 patients (2.0%). Of the remaining 2976 patients, synchronous PM were present in 148 patients (5.0%). The peritoneum was the only site of metastatic disease in 67 patients (45.6%). Patients with synchronous PM were significantly younger (median 66 vs. 71 years, *p* < 0.001), and more often had a pT4 stage (63.0% vs. 27.9%, *p* < 0.001) and pN2 stage (54.2% vs. 21.6%, *p* < 0.001) when compared to patients without synchronous PM (Table 1). Resection of the primary tumor was more often incomplete in the synchronous PM group (13.4% vs. 4.3%, *p* < 0.001).

**Table 1:** Baseline and surgical characteristics of patients who underwent resection of left-sided obstructive colon cancer, stratified for detection of synchronous peritoneal metastases

	Synchronous peritoneal metastases *N* = 148 (%)	No synchronous peritoneal metastases *N* = 2828 (%)	*P*
Sex (*N* = 2976)			0.493
Male	86/148 (58.1)	1562/2828 (55.2)	
Female	62/148 (41.9)	1266/2828 (44.8)	
Median age in years (i.q.r) (*N* = 2976)	66.0 (60.0–75.0)	71.0 (62.0–79.0)	< 0.001
Mean BMI in kg/m^2^ (SD) (*N* = 2516)	24.8 (3.9)	25.4 (4.3)	0.103
ASA score (*N* = 2947)			0.788
ASA I-II	99/145 (68.3)	1883/2802 (67.2)	
ASA III-IV	46/145 (31.7)	919/2802 (32.8)	
Comorbidity (*N* = 2961)	102/145 (70.3)	1981/2816 (70.3)	0.999
Previous abdominal surgery (*N* = 2948)	33/146 (22.6)	840/2802 (30.0)	0.057
Tumour localization (*N* = 2976)			0.487
Sigmoid	106/148 (71.6)	1956/2828 (69.2)	0.528
Descending colon	21/148 (14.2)	508/2828 (18.0)	0.242
Splenic flexure	21/148 (14.2)	364/2828 (12.9)	0.641
Tumour histology (*N* = 2927)			0.023
Adenocarcinoma	133/143 (93.0)	2660/2784 (95.5)	0.157
Mucinous tumour	6/143 (4.2)	105/2784 (3.8)	0.796
Signet-ring cell tumour	4/143 (2.8)	12/2784 (0.4)	0.006
Other	0/143 (0.0)	7/2784 (0.3)	1.000
Tumour differentiation (*N* = 1924)			0.010
Well/moderate	78/93 (83.9)	1689/1831 (92.2)	
Poor	15/93 (16.1)	142/1831 (7.8)	
Median no. of lymph nodes harvested (i.q.r.) (*N* = 2961)	16.0 (12.0–21.0)	15.0 (11.0–21.0)	0.311
Median no. of positive lymph nodes (i.q.r.) (*N* = 2958)	4.0 (1.0–9.0)	1.0 (0.0–3.0)	< 0.001
pT stage (*N* = 2960)			< 0.001
pT1	1/146 (0.7)	7/2814 (0.2)	0.333
pT2	4/146 (2.7)	104/2814 (3.7)	0.548
pT3	49/146 (33.6)	1918/2814 (68.2)	< 0.001
pT4	92/146 (63.0)	785/2814 (27.9)	< 0.001
pN stage (*N* = 2950)			< 0.001
pN0	22/144 (15.3)	1229/2806 (43.8)	< 0.001
pN1	44/144 (30.6)	970/2806 (34.6)	0.323
pN2	78/144 (54.2)	607/2806 (21.6)	< 0001
Synchronous distant metastases outside the peritoneal cavity (*N* = 2969)	80/147 (54.4)	516/2822 (18.3)	< 0.001
Liver	70/147 (47.6)	460/2775 (16.6)	< 0.001
Lung	24/144 (16.7)	102/2763 (3.7)	< 0.001
Other	17/142 (12.0)	37/2751 (1.3)	< 0.001
Initial intervention for acute colonic obstruction (*N* = 2976)			0.422
Emergency resection	121/148 (81.8)	2231/2828 (78.9)	0.404
Decompressing stoma	20/148 (13.5)	359/2828 (12.7)	0.771
SEMS without SEMS-related perforation	6/148 (4.1)	214/2828 (7.6)	0.111
SEMS with SEMS-related perforation	1/148 (0.7)	24/2828 (0.8)	1.000
Initial treatment intent			< 0.001
Curative	64/148 (43.2)	2461/2828 (87.0)	
Palliative	84/148 (56.8)	367/2828 (13.0)	
Laparoscopic approach for tumour resection (*N* = 2960)	19/147 (12.9)	454/2813 (16.1)	0.300
Conversion (*N* = 426)	5/17 (29.4)	104/409 (25.4)	0.777
Type of resection (*N* = 2975)			0.878
Sigmoid resection	98/148 (66.2)	1808/2827 (64.0)	0.576
Left hemicolectomy	38/148 (25.7)	756/2827 (26.7)	0.775
Subtotal colectomy	11/148 (7.4)	200/2827 (7.1)	0.869
Extended left hemicolectomy	0/148 (0.0)	22/2827 (0.8)	0.624
Combined sigmoid resection and right hemicolectomy	0/148 (0.0)	25/2827 (0.9)	0.633
Transverse colectomy	1/148 (0.7)	16/2827 (0.6)	0.581
Primary anastomosis (*N* = 2515)	58/148 (39.2)	1324/2817 (47.0)	0.063
Stoma in situ directly after resection (*N* = 2929)	103/144 (71.5)	1759/2785 (63.2)	0.042
Completeness of resection (*N* = 2873)			
R0	110/127 (86.6)	2628/2746 (95.7)	
R1–2	17/127 (13.4)	118/2746 (4.3)	< 0.001
Median follow-up in months (i.q.r.) (*N* = 2909)	16.0 (7.0–27.0)	27.0 (12.0–48.0)	< 0.001

*SEMS* self-expandable metal stent, *SD* standard deviation, *i.q.r.* interquartile range, *BMI* body mass index, *ASA* American Society of Anaesthesiologists

#### Treatment and Survival of Synchronous PM

**Fig. 2:**
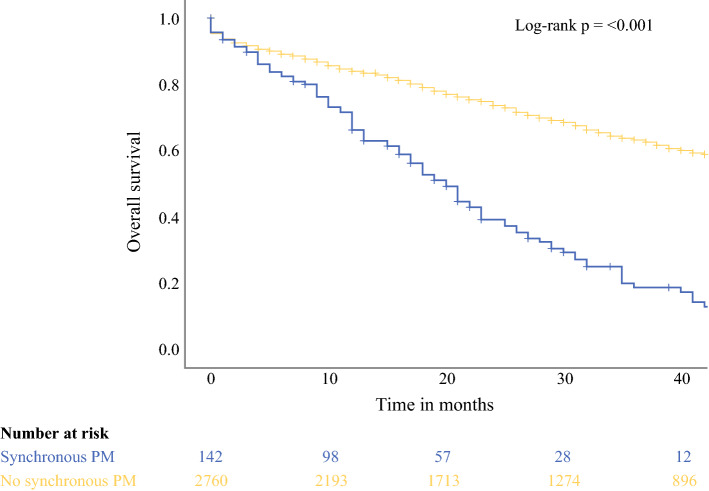
Overall survival in patients with versus without synchronous peritoneal metastases

Median follow-up of the entire cohort was 26 months (IQR 12–47). Median overall survival was 20 months (95% CI 17–23) in patients with synchronous PM and 63 months (95% CI 58–68) for patients without synchronous PM (*p* < 0.001) (Fig. 2). Three-year overall survival rates were 18.8% and 64.0%, respectively. After diagnosis of synchronous PM, 25 patients (17.0%) were treated with CRS/HIPEC.

### Metachronous Peritoneal Metastases

#### Baseline and Procedural Characteristics

Data on peritoneal recurrence were missing in 115 of 2407 patients (4.8%). Metachronous PM developed in 210 of the 2292 evaluable patients after a median interval from primary tumor resection of 14 months (IQR 9.0–22.0). The cumulative metachronous PM rate at 1, 2 and 3 years was 3.8%, 8.0%, and 9.9%, respectively. Baseline characteristics stratified for metachronous PM are displayed in Table 2. Patients with metachronous PM were significantly younger (69 vs. 71 years, *p *< 0.001), were less often ASA III-IV (22.6% vs. 30.5%, *p* = 0.017), and fewer patients had comorbidities (62.4% vs. 70.8%, *p* = 0.012). Metachronous PM patients were more often diagnosed with pT4 stage (39.7% vs. 24.7%, *p* < 0.001) and pN2 stage (29.2% vs. 17.1%, *p* < 0.001), and had a higher proportion of incomplete resection (6.0% vs. 3.3%, *p* = 0.067). More patients in the metachronous PM group were treated with adjuvant systemic chemotherapy (52.9% vs. 39.7%, *p* < 0.001).

**Table 2:** Baseline and surgical characteristics of patients who underwent curative intent resection of left-sided obstructive colon cancer, and who developed metachronous peritoneal metastases beyond 30 days postoperatively versus those who did not

	Metachronous peritoneal metastases *N* = 210 (%)	No metachronous peritoneal metastases *N* = 2082 (%)	*P*
Sex (*N* = 2292)			0.207
Male	104/210 (49.5)	1126/2082 (54.1)	
Female	106/210 (50.5)	956/2082 (45.9)	
Median age in years (i.q.r) (*N* = 2292)	69.0 (61.0–76.0)	71.0 (62.0–78.0)	< 0.001
Mean BMI in kg/m^2^ (SD) (*N* = 2008)	25.4 (23.2–28.4)	24.9 (22.7–27.6)	0.081
ASA score (*N* = 2269)			0.017
ASA I-II	161/208 (77.4)	1432/2061 (69.5)	
ASA III-IV	47/208 (22.6)	629/2061 (30.5)	
Comorbidity (*N* = 2283)	131/210 (62.4)	1467/2073 (70.8)	0.012
Previous abdominal surgery (*N* = 2272)	52/207 (25.1)	635/2065 (30.8)	0.093
Tumour localization (*N* = 2292)			0.609
Sigmoid	140/210 (66.7)	1421/2082 (68.3)	0.639
Descending colon	38/210 (18.1)	394/2082 (18.9)	0.770
Splenic flexure	32/210 (15.2)	267/2082 (12.8)	0.322
Tumour histology (*N* = 2253)			0.003
Adenocarcinoma	188/208 (90.4)	1962/2045 (95.9)	< 0.001
Mucinous tumour	16/208 (7.7)	72/2045 (3.5)	0.003
Signet-ring cell tumour	2/208 (1.0)	7/2045 (0.3)	0.199
Other	2/208 (1.0)	4/2045 (0.2)	0.099
Tumour differentiation (*N* = 1487)			0.758
Well/moderate	122/132 (92.4)	1262/1355 (93.1)	
Poor	10/132 (7.6)	93/1355 (6.9)	
Median no. of lymph nodes examined (i.q.r.) (*N* = 2286)	14.0 (11.0–20.0)	15.0 (12.0–21.0)	0.064
Median no. of positive lymph nodes (i.q.r.) (*N* = 2281)	2.0 (0.0–4.0)	1.0 (0.0–2.0)	< 0.001
pT stage (*N* = 2282)			< 0.001
pT1	1/209 (0.5)	5/2073 (0.2)	0.438
pT2	5/209 (2.4)	86/2073 (4.1)	0.216
pT3	120/209 (57.4)	1471/2073 (71.0)	< 0.001
pT4	83/209 (39.7)	511/2073 (24.7)	< 0.001
pN stage (*N* = 2277)			< 0.001
pN0	72/209 (34.4)	1015/2068 (49.1)	< 0.001
pN1	76/209 (36.4)	700/2068 (33.8)	0.465
pN2	61/209 (29.2)	353/2068 (17.1)	< 0.001
Synchronous distant metastases outside the peritoneal cavity (*N* = 2241)	23/208 (11.1)	161/2033 (7.9)	0.116
Liver	22/208 (10.6)	138/2030 (6.8)	0.044
Lung	1/208 (0.5)	19/2030 (0.9)	1.000
Other	0/208 (0.0)	15/2028 (0.7)	0.387
Initial intervention for acute colonic obstruction (*N* = 2292)			0.802
Emergency resection	169/210 (80.5)	1602/2082 (76.9)	0.245
Decompressing stoma	25/210 (11.9)	291/2082 (14.0)	0.406
SEMS without SEMS-related perforation	15/210 (7.1)	174/2082 (8.4)	0.542
SEMS with SEMS-related perforation	1/210 (0.5)	15/2082 (0.7)	1.000
Laparoscopic approach for tumour resection (*N* = 2280)	33/209 (15.8)	347/2071 (16.8)	0.721
Conversion (%) (*N* = 339)	6/32 (18.8)	80/307 (26.1)	0.366
Type of resection (*N* = 2291)			0.238
Sigmoid resection	121/210 (57.6)	1324/2081 (63.6)	0.087
Left hemicolectomy	65/210 (31.0)	582/2081 (28.1)	0.376
Subtotal colectomy	19/210 (9.0)	131/2081 (6.3)	0.124
Extended left hemicolectomy	1/210 (0.5)	18/2081 (0.9)	1.000
Combined sigmoid resection and right hemicolectomy	2/210 (1.0)	16/2081 (0.8)	0.678
Transverse colectomy	2/210 (1.0)	8/2081 (0.4)	0.232
Primary anastomosis (*N* = 2283)	94/209 (45.0)	1014/2074 (48.9)	0.280
Stoma in situ directly after resection (*N* = 2255)	135/207 (65.2)	1263/2048 (61.7)	0.316
Completeness of resection (%) (*N* = 2237)			0.067
R0	189/201 (94.0)	1969/2036 (96.7)	
R1–2	12/201 (6.0)	67/2036 (3.3)	
Adjuvant chemotherapy (*N* = 2286)	110/208 (52.9)	826/2078 (39.7)	< 0.001
Median time in weeks from resection until start adjuvant chemotherapy (i.q.r.) (*N* = 838)	6.0 (4.0–11.0)	6.0 (4.0–8.0)	0.435
Median follow-up in months (i.q.r.) (*N* = 2245)	26.0 (16.5–39.0)	32.0 (16.0–54.0)	0.006

*SEMS* self-expandable metal stent, *SD* standard deviation, *i.q.r.* interquartile range, *BMI* body mass index, *ASA* American Society of Anaesthesiologists

#### Risk Factors for the Development of Metachronous PM in LSOCC

Univariable analysis revealed the following potential predictors of metachronous PM: subtotal colectomy, pT4 stage, pN1 stage, pN2 stage, incomplete (R1–2) resection, having received adjuvant chemotherapy, and time from resection until adjuvant chemotherapy of ≥ 8 weeks (Table 3). Subsequent multivariable analysis identified only pT4 stage (HR 1.78, 95% CI 1.19–2.67, *p *= 0.005) and pN2 stage (HR 2.10, 95% CI 1.21–3.65, *p* = 0.009) as independent risk factors.

**Table 3:** Cox proportional hazards regression analysis of risk factors for developing metachronous peritoneal metastases after resection of left-sided obstructive colon cancer

Variable	Univariable analysis		Multivariable analysis	
	HR (95% CI)	*P*	HR (95% CI)	*P*
Age				
< 60 years	Reference			
≥ 60 years	0.905 (0.651–1.259)	0.554	–	–
ASA score				
ASA 1–2	Reference			
ASA 3–4	0.828 (0.598–1.147)	0.257	–	–
Treatment				
Emergency resection	Reference			
Elective resection after DS or SEMS without perforation	0.809 (0.571–1.146)	0.232	–	–
SEMS with perforation	0.651 (0.091–4.653)	0.669		
Surgical approach				
Open	Reference			
Laparoscopic	0.932 (0.639–1.358)	0.713	–	–
Type of resection				
Segmental resection	Reference			
Subtotal colectomy	1.597 (0.996–2.560)	0.052	NS	NS
Major post-resection complications				
No	Reference			
Yes	0.933 (0.627–1.390)	0.735	–	–
Tumour histology				
Non-mucinous	Reference			
Mucinous	2.383 (1.430–3.973)	0.001	NS	NS
pT stage				
pT1–3	Reference		Reference	
pT4	2.176 (1.646–2.876)	< 0.001	1.782 (1.191–2.668)	0.005
pN stage				
pN0	Reference		Reference	
pN1	1.557 (1.127–2.152)	0.007	1.207 (0.696–2.094)	0.503
pN2	2.599 (1.844–3.662)	< 0.001	2.101 (1.208–3.653)	0.009
Location of tumour				
Splenic flexure	Reference		–	–
Descending colon	0.848 (0.528–1.361)	0.495		
Sigmoid	0.842 (0.573–1.236)	0.379		
Radicality				
R0	Reference			
R1–2	2.141 (1.194–3.840)	0.011	NS	NS
Adjuvant chemotherapy				
No	Reference			
Yes	1.389 (1.056–1.825)	0.019	NS	NS
Time from resection until adjuvant chemotherapy				
< 8 weeks	Reference			
≥ 8 weeks	1.314 (0.874–1.975)	0.189	NS	NS

*ASA* American Society of Anaesthesiologists, *DS* decompressing stoma, *SEMS* self-expandable metal stent, *NS* not significant

#### Treatment and Survival of Metachronous PM

**Fig. 3:**
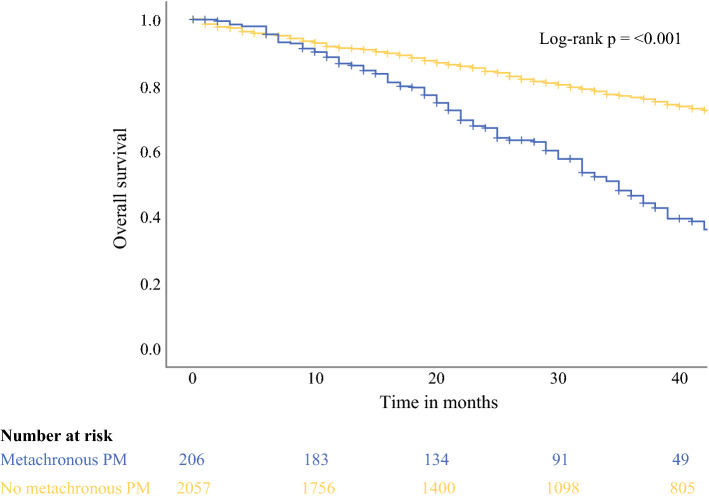
Overall survival in patients who did or did not develop metachronous peritoneal metastases as measured from time of primary tumour resection

Treatment of metachronous PM was judged by the local investigators as intentionally curative in 59 patients (28.6%) (Supplementary Table 1), and this consisted of CRS/HIPEC in 41 patients (19.9%). A total of 147 patients (71.4%) were treated with palliative intent, mostly comprising palliative systemic therapy. Median follow-up was 31 months (IQR 15–52). Three-year overall survival was 48.1% and 77.0% for patients developing metachronous PM and patients who did not, respectively (*p* < 0.001) (Fig. 3).

## Discussion

The present population based analysis of patients who underwent resection of LSOCC revealed an incidence of synchronous PM of 5%. The cumulative 3-year metachronous PM rate of the remaining patients who were treated with curative intent and were still alive at 30 days postoperatively was 9.9%. Within this clearly defined cohort of colon cancer patients who present with an emergency obstruction, pT4 and pN2 stage appeared to be independent risk factors for developing metachronous PM. A substantial prognostic impact of both synchronous and metachronous PM could be demonstrated. In both the synchronous and metachronous PM groups, less than 20% were eligible for surgical treatment of PM.

There are a few other population based studies on incidence of synchronous PM of colorectal origin. Another Dutch study from the Eindhoven Cancer registry found an incidence of 4.8% among 18,738 patients diagnosed with primary colorectal cancer between 1995 and 2008, of whom 44% had metastatic disease limited to the peritoneal cavity.^13^ The definition of synchronous PM was not provided and no data were given on emergency presentation. This incidence was confirmed at a national level by Van der Geest et al., reporting a 4.7% synchronous PM rate between 2008 and 2011.^14^ Synchronous PM were found in 477 of 11,124 colorectal cancer patients (4.3%) in the Stockholm region (1995–2007) within 1 month from diagnosis.^6^ This study did not provide separate data on emergency surgery or obstruction. A recent study based on the entire country of Sweden, including 35,120 colorectal and appendiceal cancers surgically treated between 2007 and 2015, reported a 2.5% incidence of synchronous PM within 6 months from diagnosis.^7^ Perforation close to a colon cancer was only statistically significant in univariable analysis, but emergency surgery for colon cancer remained independently associated with synchronous PM in multivariable analysis. An overall incidence of synchronous PM of 6.8% was reported in a French study including 9148 colorectal cancer patients (1976–2011) from the administrative area of Côte-d’Or in Burgundy, also using a 6-month period from diagnosis of the primary cancer. Among 737 patients with obstructing colorectal cancer, the incidence of synchronous PM was 16.2%, with a corresponding odds ratio for obstruction of 2.8 when compared with non-emergency surgery in univariable analysis.^15^

The present cohort represents a certain subpopulation with only left-sided obstructing tumors. Left-sided location has been associated with lower risk of PM, whereas emergency setting and the more advanced T as well as N stage would imply a higher risk of PM if compared to an unselected colon cancer population. The 5.0% observed incidence of synchronous PM in the current study is difficult to compare with the available literature, given the varying definitions (PM diagnosed within 1–6 months), populations and time periods that were included. Using the same definition regarding diagnosis within 1 month from primary resection, the incidence of the current population with obstruction is probably only slightly higher if compared with the unselected population of Segelman et al.^6^

Reported survival of synchronous PM based on registry data is generally poor, ranging from a median survival of 5 months in older studies and in combination with other metastatic sites, up to a 3-year overall survival rate of 21% with surgical treatment in more recent years.^13^^,^^15^ Besides surgical treatment, mostly consisting of CRS/HIPEC, the use of systemic therapy has also substantially increased over the years, which translated into better survival at a population level.^15^^,^^16^

The 3-year metachronous PM rate of 9.9% as found in our selected population constituting left-sided obstructing colon cancer, seems substantially higher compared to unselected colon cancer populations described in the literature. A recent pooled analysis of three large randomized trials on adjuvant treatment after curative resection of stage II-III colon cancer revealed an overall crude incidence of only 2.3% (86/3714).^17^ Younger age (< 60 years), pT4, pN1–2 and D2 (instead of D3) lymphadenectomy were found to be independent predictors for metachronous PM, while adjuvant chemotherapy, mucinous histology and differentiation were not associated. The authors explain the relatively low rate of metachronous PM by the fact that no perforated tumors were included in these trials, with very low rates of obstruction, emergency surgery and incomplete resection.

A previous Dutch population based study reported a crude incidence of metachronous PM of 3.4% (197/5671).^18^ No data on emergency setting was available. One of the reasons for the higher percentage of PM in the present study might be the fact that this is a more recent cohort of patients. Over the years, the use of CT imaging during follow-up has intensified and the quality of CT imaging has improved. A similar 4.2% metachronous PM rate was found in the previously mentioned study from Stockholm County.^6^ Emergency surgery was associated with higher risk of metachronous PM. Although reasons for emergency surgery were not reported, bowel perforation was separately included in the multivariable model, and did not show a significant association. The more recent data from Sweden confirmed that perforation was not associated with metachronous PM, while emergency surgery for colon cancer was independently associated with metachronous PM (HR 1.92).^7^ The French study by Quere et al. reported an overall cumulative incidence of metachronous PM of 5.5% at 5 years.^15^ Obstruction or perforation were both associated with a 10% and 12% cumulative risk of metachronous PM at 3 and 5 years, respectively, with a corresponding combined HR of 1.82 in multivariable analysis. Our study confirms these observations and supports the hypothesis that obstruction is an independent risk factor for metachronous PM.

Remarkably, observed HR for T4 of 1.8 and for N2 of 2.1 in the present study are much lower than in the published multivariable models. Segelman et al. developed a prediction model for colon and rectal cancer separately, which was subsequently validated.^19^^,^^20^ Right-sided tumor location (HR 1.23), number of harvested lymph nodes < 12 (HR 1.64), R1 resection (HR 1.49), R2 resection (HR 2.31) and emergency surgery (HR 2.09) were of minor influence, whereas the highest risk was observed for T4 (HR 19.44) and N2 stage (HR 4.51). External validation resulted in modification of the model with incorporation of mucinous histology, but still showing the two dominant predictors of T4 and N2 stage. The relatively low HRs as found in the present analysis are likely explained by a higher a priori risk, mainly caused by more advanced T and N stage at baseline. This might also support the finding of the French study that obstruction itself increases the risk of metachronous PM, after which the impact of TN stage is reduced.

The literature suggests that more intensified surgical and systemic treatment in selected patients with metachronous PM results in favourable survival.^15^^,^^16^ However, the proportion of patients that are eligible for multimodality treatment including surgery is still restricted based on the present study, emphasizing the need for studies that aim for optimized detection and management of metachronous PM.^21^

Limitations of the present study are related to the retrospective data collection and some degree of missing data. For example, treatment of synchronous PM was registered inconsistently with consequent limited information. Furthermore, the relative contribution of obstruction to the risk of synchronous or metachronous PM could not be determined because of the lack of a control group. Another important limitation is the difficulty of diagnosing PM with standard imaging techniques. For example, the incidence of PM in CRC patients undergoing post-mortem autopsy is even up to 40%, depending on tumour type (mucinous and signet ring cell vs. adenocarcinoma) and location.^22^ Nevertheless, this study adds to the currently available literature by providing up to date epidemiological data on peritoneal dissemination in patients with LSOCC.

In conclusion, this population based study revealed a 5.0% incidence of synchronous peritoneal metastases in patients who underwent resection of left-sided obstructive colon cancer. The subsequent 3-year cumulative metachronous PM rate was 9.9%, with advanced tumour and nodal stage as independent risk factors for the development of PM. This relatively high rate supports the literature suggesting that obstruction is independently associated with an increased risk of metachronous PM.

## Electronic supplementary material

Below is the link to the electronic supplementary material.

Supplementary material 1 (DOCX 12 kb)
