# Supplementary material for: Synchronous and Metachronous Peritoneal Metastases in Patients with Left-Sided Obstructive Colon Cancer
Source: Ann Surg Oncol. 2020 Mar 13;27(8):2762–73. doi: 10.1245/s10434-020-08327-7 (PMC7334250; doi:10.1245/s10434-020-08327-7)
Supplement: Supplementary file 1 — Supplementary material 1 (DOCX 12 kb) [file 10434_2020_8327_MOESM1_ESM.docx]

**Supplementary Table 1.** Treatment of metachronous peritoneal metastases.

| **Treatment** | **Metachronous peritoneal metastases N = 210 (%)^a^** |
| --- | --- |
| **Curative intent**  Cytoreductive surgery + HIPEC  Resection +/- perioperative systemic therapy  Other | 59/206 (28.6) 41/206 (19.9) 17/206 (8.3) 1/206 (0.5) |
| **Palliative intent** Palliative systemic therapy  Palliative surgery (bypass/stoma, etc.)  Best supportive care  Other  Unknown | 147/206 (71.4) 72/206 (35.0) 10/206 (4.9) 54/206 (26.2) 4/206 (1.9) 7/206 (3.4) |

HIPEC = hyperthermic intraperitoneal chemotherapy
^a^ Of 210 patients, treatment intent was unknown in 4 patients
